# An RNA Transport System in *Candida albicans* Regulates Hyphal Morphology and Invasive Growth

**DOI:** 10.1371/journal.pgen.1000664

**Published:** 2009-09-25

**Authors:** Sarah L. Elson, Suzanne M. Noble, Norma V. Solis, Scott G. Filler, Alexander D. Johnson

**Affiliations:** 1 Department of Microbiology and Immunology, University of California San Francisco, San Francisco, California, United States of America; 2 Department of Medicine, Division of Infectious Diseases, University of California San Francisco, San Francisco, California, United States of America; 3 Los Angeles Biomedical Research Institute at Harbor-UCLA Medical Center, Torrance, California, United States of America; 4 David Geffen School of Medicine at UCLA, Los Angeles, California, United States of America; 5 Department of Biochemistry and Biophysics, University of California San Francisco, San Francisco, California, United States of America; Fred Hutchinson Cancer Research Center, United States of America

## Abstract

Localization of specific mRNAs is an important mechanism through which cells achieve polarity and direct asymmetric growth. Based on a framework established in *Saccharomyces cerevisiae*, we describe a She3-dependent RNA transport system in *Candida albicans*, a fungal pathogen of humans that grows as both budding (yeast) and filamentous (hyphal and pseudohyphal) forms. We identify a set of 40 mRNAs that are selectively transported to the buds of yeast-form cells and to the tips of hyphae, and we show that many of the genes encoded by these mRNAs contribute to hyphal development, as does the transport system itself. Although the basic system of mRNA transport is conserved between *S. cerevisiae* and *C. albicans*, we find that the cargo mRNAs have diverged considerably, implying that specific mRNAs can easily move in and out of transport control over evolutionary timescales. The differences in mRNA cargos likely reflect the distinct selective pressures acting on the two species.

## Author Summary

Generation of cellular polarity – asymmetry in shape, protein distribution, and/or sub-cellular function – is an essential feature of most eukaryotic cells and underlies such diverse processes as differentiation, mating, nutrient acquisition, and growth. Localization of specific mRNAs is one mechanism through which cells achieve polarity. We describe an RNA transport system in *Candida albicans*, a fungal pathogen of humans, that grows in both single cell (budding yeast) and filamentous (hyphal and pseudohyphal) forms. Hyphae are chains of elongated cells that remain attached after cell division and exhibit highly polarized growth at their tips. We show that the *C. albicans* She3-dependent RNA transport system binds to 40 mRNAs and transports these mRNAs to yeast buds and to the tips of hyphae. Both the transport system itself and many of the genes encoded by transported mRNAs are required for normal growth and function of hyphae. Although the basic transport mechanism appears conserved with that of the model yeast, *Saccharomyces cerevisiae*, the cargo mRNAs are largely distinct. The apparently rapid evolution of the transported mRNAs probably reflects distinct selective pressures acting on the two organisms.

## Introduction

Cell polarity – asymmetry in shape, protein distribution, and/or sub-cellular function – is an essential feature of most eukaryotic cells and underlies such fundamental processes as cell division, cell differentiation, and cell-cell communication. One mechanism for achieving cellular asymmetry is through the localization of specific mRNAs to different parts of the cell. For instance, the spatial distribution of specific mRNAs in the oocytes of *Drosophila melanogaster* and *Xenopus laevis* underlies establishment of embryo polarity in these organisms [1],[2],[3],[4],[5]. In chick fibroblasts, transport of beta-actin mRNA promotes actin assembly at the leading edge of the cells [6],[7],[8], and in mammalian neurons, transport of RNA to dendrites for localized protein synthesis is critical to synaptic activity [9],[10],[11],[12]. In each of these examples, RNA localization occurs via active transport along cytoskeletal elements: microtubules in the *Drosophila* embryo, microfilaments in chick fibroblasts, and both structures in the *Xenopus* embryo and in mammalian neurons.

Selective RNA transport is also a key feature of fungi. In the maize pathogen *Ustilago maydis*, the Rrm4 protein binds RNA and moves along microtubules. Loss of Rrm4, or mutation of its RNA-binding domain, results in polarity defects and reduced virulence of the organism [13],[14]. One of the best understood RNA localization mechanisms is the *Saccharomyces cerevisiae* She system, a riboprotein complex that uses actomyosin transport to move a set of mRNAs from the mother cell to the bud during mitosis [15],[16],[17],[18],[19]. Within the She complex, She2 is thought to be the primary RNA binding protein that links specific mRNAs to Myo4, a type V myosin motor, via the adaptor protein She3 [20],[21],[22],[23]. Thus, a small set of mRNAs, selected by binding to She2, is transported from the mother cell to the bud. One such mRNA encodes Ash1, a transcriptional repressor of HO, an endonuclease required for mating-type interconversion; Ash1 localization to daughter cells ensures that only mother cells express HO and thereby undergo this type of programmed DNA rearrangement [24],[25],[26],[27].

In this study, we investigated the biological role of She-dependent RNA transport in *Candida albican*s, a commensal fungus and an opportunistic pathogen that can cause severe infection in immunocompromised humans. In the host, *C. albicans* exists in a variety of morphological forms, including budding yeast, pseudohyphae (chains of elongated ellipsoidal cells), and hyphae (chains of long, cylindrical cells with parallel cell walls) [28]. The ability to rapidly switch among these forms in response to external cues is one of numerous factors contributing to virulence. The hyphal form in particular has been associated with numerous virulence attributes such as passage through endothelial and epithelial barriers and host tissue damage.

*C. albicans* hyphae are formed by polarized growth at the apical cell (the hyphal tip cell). Several morphological and molecular characteristics distinguish the hyphal tip cell from the sub-apical (*i.e.*, non-tip) cells of the filament. Newly formed apical cells inherit most of the cytoplasm and are cytologically active, while the mother or sub-apical cells are extensively vacuolated and undergo temporary cell cycle arrest [29]. Further, the Golgi complex is continuously redistributed to tip cells [30], suggesting a means by which hyphae achieve localized secretion at their tips. As in other filamentous fungi, the tip of *C. albicans* hyphae contains the Spitzenkörper, a cluster of exocytic vesicles that drives polarized growth by concentrating secretion at the tip [31]. Finally, there is evidence that hyphal tip cells serve a specialized function during *C. albicans* invasion of host tissues. Electron micrographs have shown a zone of clearing around hyphae penetrating mammalian epithelia, suggesting a concentration of hydrolytic enzymes at the invading tip [32]. At least one such enzyme, phospholipase B, has been shown to be preferentially secreted from the hyphal tip cells [33].

In this study, we establish the existence of a She3-dependent mRNA transport system in *C. albicans*. In addition, we (1) identify a set of RNA transcripts specifically bound to She3; (2) determine the cellular localization of She3-bound transcripts; (3) characterize the phenotypes associated with loss of She3, and (4) study the effects of deleting individual genes whose mRNAs are She3-bound. From the results of these experiments, we conclude that *C. albicans* has a She3-mediated system that transports selected transcripts into both daughter cells of budding yeast and into tip cells of the hyphae. We further show that approximately one third of these transcripts have roles in hyphal development. Finally, we show that She-based RNA transport, although not required for hyphal growth *per se*, is important for proper hyphal morphology and for specific aspects of hyphal function, specifically, invasive hyphal growth and tissue damage.

Although the general features of the *C. albicans* She transport system appear conserved with those of *S. cerevisiae*, the mRNAs carried by She3 differ considerably between the two species, suggesting relatively rapid evolutionary turnover in the set of cargo mRNAs. This finding is analogous to comparisons of transcriptional circuits between *C. albicans* and *S. cerevisiae*; although the transcriptional regulators are often highly conserved, the genes they regulate can differ considerably.

## Results

### Actin-based RNA transport in *C. albicans*

A search of the genome sequence of *C. albicans* (www.candidagenome.org) revealed ORF19.5595, predicted to encode a 377 amino acid protein, as a likely ortholog of *S. cerevisiae SHE3*. An alignment of this protein with other putative fungal She3 orthologs indicates that the region of highest conservation is in the amino-terminal half of the protein, the putative myosin interaction domain [23]. No clear *SHE2* ortholog was identified in *C. albicans*; either She3 may serve as the RNA-binding protein, or another, yet-unidentified protein may fulfill this function in *C. albicans*. The *C. albicans* genome contains a single gene encoding a class V myosin, *MYO2* (orf19.5015 [34]); if the RNA transport mechanisms are similar in *S. cerevisiae* and *C. albicans*, Myo2 is most likely the motor linking She3 to actin filaments.

Previous work has supported the idea that a She3-dependent mechanism of RNA transport may operate in *C. albicans*. *C. albicans* Ash1 protein is restricted to the tip cells of hyphae, as well as to daughter cells of budding yeast [35]. When the mRNA encoding *C. albicans* Ash1 was expressed in *S. cerevisiae*, it accumulates in daughter cells [36], indicating that the *C. albicans ASH1* transcript may contains localization signals that are recognized by the *S. cerevisiae* She complex.

**Figure 1:**
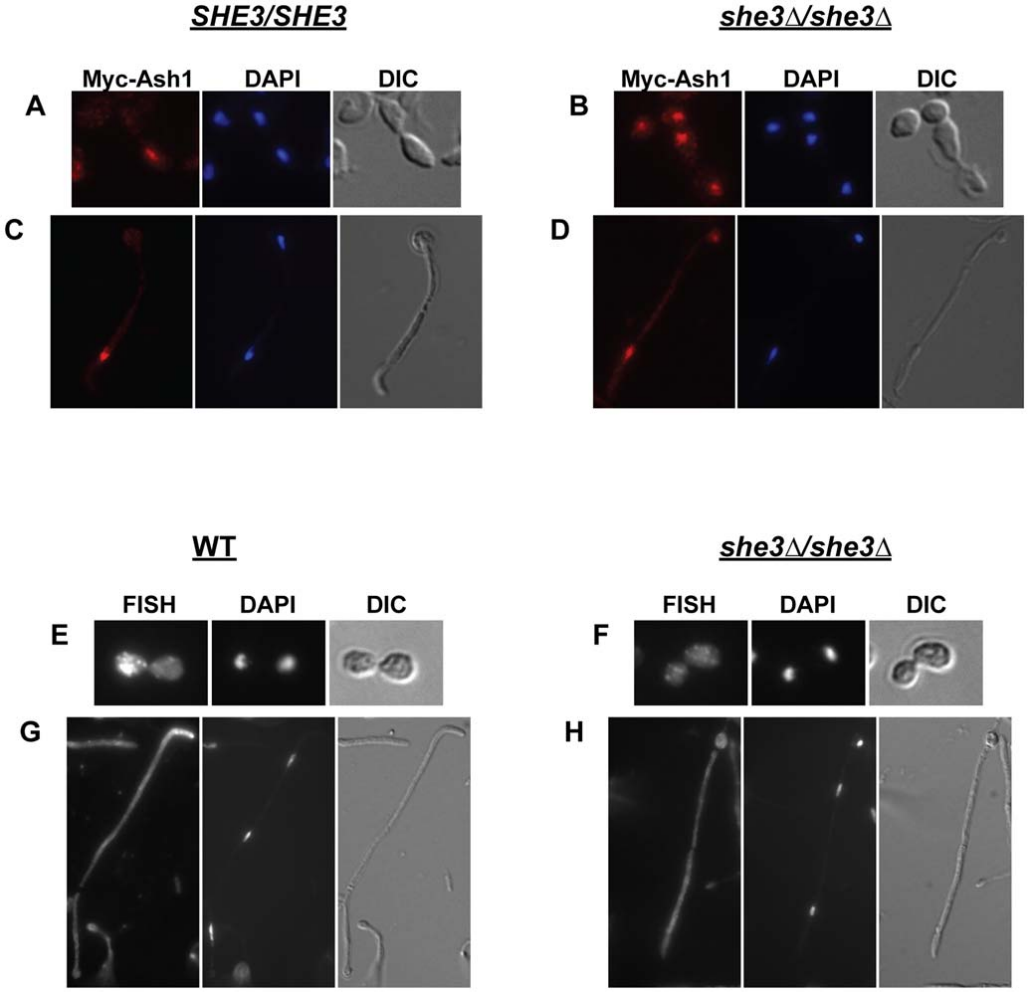
Ash1 protein and *ASH1* mRNA are mis-localized in *C. albicans* lacking She3. (A–D) *SHE3/SHE3* [SE18, (A,C)] and *she3Δ/she3Δ* cells [SE20, (B,D)] carrying a myc-tagged version of Ash1 (Myc-Ash1) were processed for indirect immunofluorescence, as described [35]. Cells were stained with the mouse 9E10 anti-myc antibody followed by the Alexa-546 secondary antibody (red). Cell nuclei were visualized with DAPI (blue). In a wild type background, Myc-Ash1 accumulates in daughter cells of yeast (A) and in tip cells of hyphae (C). In the *she3Δ/she3Δ* strain, myc-Ash1 accumulates in both mother and daughter cells of yeast (B) and in tip and non-tip cells of hyphae (D). (E–H) Cells from wild type (“WT,” CAF2-1) and *she3Δ/she3Δ* (SE4) strains were processed for fluorescent *in situ* hybridization (FISH) to detect endogenous *ASH1* transcript; cell nuclei were visualized with DAPI. Probe signal accumulates in the daughter cell of wild-type *C. albicans* yeast (E) and in the tips of hyphae (G). There is no specific localization of probe signal in yeast or in hyphae lacking She3 (F,H).

To directly test whether *C. albicans* possesses a She3-dependent RNA transport system, we deleted both copies of the *C. albicans SHE3* gene (*C. albicans* is diploid) (strains used in this study are listed in Table 1). We observed that Ash1 now appears in both mother and daughter nuclei in yeast, and in nuclei of multiple cells of hyphae (Figure 1). We used fluorescent *in situ* hybridization (FISH) to detect localization of the endogenous *ASH1* transcript in wild type and *she3Δ/she3Δ* cells. We observed that *ASH1* mRNA accumulates in yeast daughter cells and in the tips cells of hyphae in a She3-dependent manner. The results indicate that *C. albicans* Ash1 localization (to daughter cells in yeast and to tip cells in hyphae) is mediated by She3 and likely occurs through specific localization of the *ASH1* transcript – as occurs in *S. cerevisiae*.

**Table 1:** Names and genotypes of strains used in this study.

Strain Name	Genotype	Reference
CAF2-1	*URA3/ura3::imm434*	[51]
QMY23	*leu2::pLEU2/leu2::pHIS1; his1Δ/his1Δ*	[52]
SE4	*she3Δ/she3::URA3; ura3Δ/ura3Δ*	This study
SE5	*she3Δ/she3Δ; ura3Δ/ura3Δ*	This study
SE6	*she3Δ/SHE3; ura3Δ/ura3Δ*	This study
SE18	*ash1::p6MYC-ASH1::URA3/ASH1; ura3Δ/ura3Δ*	This study
SE20	*ash1::p6MYC-ASH1::URA3/ASH1; she3Δ/she3Δ; ura3Δ/ura3Δ*	This study
SE25	*SHE3-TAP::URA3/she3Δ; ura3Δ/ura3Δ*	This study
SE28	*she3::HIS1/SHE3; leu2Δ/leu2Δ; his1Δ/his1Δ; arg4Δ/arg4Δ*	This study
SE30	*she3::HIS1/she3::LEU2; leu2Δ/leu2Δ; his1Δ/his1Δ*	This study
SE32	*she3::HIS1/she3::LEU2; leu2Δ/leu2Δ; his1Δ/his1Δ; arg4Δ/arg4Δ*	This study
SE61	*she3::HIS1/SHE3; leu2Δ/leu2Δ; his1Δ/his1Δ; arg4Δ/arg4Δ; RPS1-pARG4*	This study
SE63	*she3::HIS1/she3::LEU2; leu2Δ/leu2Δ; his1Δ/his1Δ; arg4Δ/arg4Δ; RPS1-pARG4*	This study
SE64	*she3::HIS1/she3::LEU2; leu2Δ/leu2Δ; his1Δ/his1Δ; arg4Δ/arg4Δ; RPS1-pSHE3-ARG4*	This study

### Identification of She3-associated RNAs

We used immunoprecipitation (IP) of She3-RNA complexes, followed by hybridization to whole genome microarrays, to identify the set of RNAs bound and potentially localized by *C. albicans* She3 (Figure S1). Cellular lysates were prepared from a *C. albicans* strain (YSE25) containing a single copy of She3 fused to a tandem affinity purification tag (She3-TAP) [37], which was grown in the yeast form (YEPD medium 30°C) or induced to form hyphae by addition of serum at 37°C for 30 minutes, one hour, or three hours. The tagged She3 protein was immunoprecipitated from these lysates, and the associated RNAs were eluted. Labeled cDNA generated from the She3-associated RNA was compared to reference cDNA by competitive hybridization to *C. albicans* microarrays representing the entire genome [38]. We used two different types of reference RNA: (1) total RNA from the She3-TAP strain, or (2) RNA isolated from a mock IP performed with an untagged strain. Use of the first type of reference risks false positives inherent to the IP methods (*i.e.*, “sticky RNAs”), whereas use of the second is subject to potential complications arising from the use of two different strains. In a given experiment, we used either one reference or the other, and we combined results for data analysis, as explained below. This approach allowed us to eliminate false positives inherent to either method.

Stringent filter criteria were used to identify the set of candidate She3-associated RNAs. Data were derived from twelve microarrays from yeast (six each using either of the two reference samples) and 24 from hyphae (from each of three time points, four arrays each using the two reference populations). To pass the initial filter, array elements (spots) must have produced interpretable hybridization in greater than 50% of arrays from any single experiment (*i.e.*, from one growth condition using one reference population) and must have had a median percentile rank of at least 98. A second filter required that transcripts be identified using both reference populations, and, for those identified from hyphal lysates, be identified in at least two time points. These criteria identified a set of 31 high-confidence transcripts bound by She3 in yeast and a largely overlapping set of 38 high-confidence transcripts bound by She3 in hyphae (Table 2).

**Table 2:** Genes whose transcripts were identified as She3-associated from RNA IP experiments.

Systemic Name	Standard Name	*S. cerevisiae* Ortholog or Best Hit	Predicted Function	Predicted Biological Process	Growth Stage(s)						
						Serum Induction					
					Y	30 m	1 hr	3 hr			
orf19.3356		ESP1	Endopeptidase (separase)	Mitosis	X				X	X	X
orf19.122	CDC20	CDC20	Activator of the anaphase promoting complex	Mitosis	X				X	X	X
orf19.267		NET1	Core subunit of the RENT complex	Nucleolar regulation, mitosis	X				X	X	X
orf19.3071		MIH1	Tyrosine phosphatase	Mitosis	X				X	X	X
orf19.3153	MSS4	MSS4	1-phosphatidylinositol-4-phosphate 5-kinase	Actin cytoskeleton	X				X	X	X
orf19.6044		MOB2	Protein kinase activator, component of the RAM signaling network	Polarity						X	X
orf19.6705		YEL1	Guanyl-nucleotide exchange factor activity	Polarity					X	X	X
orf19.3895	CHT2	CTS1	Endochitinase	Cytokinesis	X				X	X	X
orf19.5537		WSC2	Stress receptor	Cell wall maintenance	X				X	X	X
orf19.207	PGA55	YMR317W	Putative GPI-anchored cell surface protein	Unknown	X				X	X	X
orf19.5224		PKH1	Serine/threonine kinase	Endocytosis,and cell wall maintenance	X				X	X	
orf19.3618	YWP1	FLO1	Putative GPI-anchored cell wall and secreted protein	Adhesion					X	X	
orf19.4765	PGA6	CCW12	Putative GPI-anchored cell surface protein	Unknown	X				X	X	X
orf19.2685	PGA54		Putative GPI-anchored cell wall protein	Unknown	X				X	X	X
orf19.6948	CCC1	CCC1	Putative manganese transporter	Cellular ion homeostsis	X				X	X	X
orf19.1536		ZRC1	Iintracellular ion transporter	Iintracellular ion homeostsis	X				X	X	X
orf19.1582		HOL1	Ion Transporter	Ion Transport					X	X	X
orf19.5406		PSR1	Plasma membrane associated protein phosphatase	Regulation of sodium ion transport	X				X	X	
orf19.5343	ASH1	ASH1	Transcription factor	Filamentous growth and virulence	X				X		X
orf19.2423	ZCF11	YBR239c	Transcription factor	Filamentous growth	X				X	X	X
orf19.3315	CTA9		Transcriptional activator	Unknown	X				X	X	X
orf19.723	BCR1	USV1	Transcription factor	Biofilm regulation	X				X	X	X
orf19.2432	HAC1	HAC1	Transcription factor	Unfolded protein response					X	X	X
orf19.3912	GLN3	GLN3	Transcription factor	Unknown	X						
orf19.6202	RBT4	PRY1	Unknown secreted protein	Virulence	X	X	X	X	X	X	X
orf19.5585*	SAP5	YPS3	Secreted protease	Virulence					X		X
orf19.3707	YHB1	YHB1	Flavohemoglobin	Nitric oxide response	X				X	X	
orf19.4432		KSP1	Serine/threonine kinase	Unknown	X				X	X	X
orf19.2219	ORF298	YIL082W-A	Transposable element genes	Unknown	X				X	X	X
orf19.5710		NSP1	Nuclear pore structural protein	Nuclear import and export					X	X	X
orf19.7228		GMH1	Golgi membrane protein	Unknown	X						
orf19.2459			Unknown	Unknown	X				X	X	X
orf19.660			Unknown	Unknown	X				X	X	X
orf19.6660		YHR131C	Unknown	Unknown	X				X	X	X
orf19.3460			Unknown	Unknown	X					X	X
orf19.5282			Unknown	Unknown	X				X	X	X
orf19.2431			Unknown	Unknown					X		X
orf19.1354		YER067W	Unknown	Unknown						X	X
orf19.3793			Unknown	Unknown	X				X	X	X
orf19.3422	FMP27	YLR454W	Unknown	Unknown	X					X	

Systemic name, standard name, and *S. cerevisiae* ortholog or best-hit are as listed in the *Candida* Genome Database (CGD, www.candidagenome.org). Predicted function and predicted biological process summarize information in the “Description,” “Molecular Function,” and “Biological Process” fields in CGD and/or the *Saccharomyces* Genome Database (www.yeastgenome.org). Growth stage(s) refers to the lysates from which transcripts were identified as She3-associated. An X indicates that at least one array element representing a given transcript passed the initial filter, as described in Results. Raw enrichment values (ratio of the medians of She3-associated RNA compared to reference) are provided in Table S3, and raw microarray data are provided in Table S4. We note that certain genes whose expression has been described as yeast- or hyphal-specific were identified in both yeast and hyphal lysates. Our IP methodology relied solely on differential She3 binding and should not require high levels of expression of bound transcripts.

*: The arrays spots for *SAP4* (orf19.5716), *SAP5* (orf19.5585), and *SAP6* (orf19.5542) cross-hybridized. In order to distinguish which of these three *SAP* transcripts were She3-associated, we performed quantitative PCR using primers specific to each *SAP* gene. cDNA from the She3-TAP RNA IP from 30 minute and three hour serum inductions was compared to cDNA from total RNA or mock IP. These experiments showed consistent enrichment of *SAP5* in the She3-TAP IP and more variable enrichment of *SAP4* and *SAP6*.

The genes represented by the set of She3-bound transcripts act in a variety of cellular processes, including mitosis and cytoskeletal dynamics, cell polarity, transcription, small molecule transport and regulation, virulence, and cell wall structure and function (Table 2). Ten genes encode proteins of unknown function. *ASH1* was identified as She3-associated in both yeast and hyphae, validating the approach and providing independent evidence that Ash1 protein is localized via She3-dependent transport of *ASH1* RNA. For the most part, the She3-bound mRNAs are the same in yeast and in hyphae; only two of these transcripts were identified as She3-bound solely in yeast, and nine were identified as bound only in hyphae. In general, these patterns do not reflect the relative abundance of the transcripts in yeast versus hyphae, as determined by previous studies (www.candidagenome.org).

### Comparison of She-associated transcripts in *S. cerevisiae* and *C. albicans*

Of the 24 RNAs identified as She-transported in *S. cerevisiae*, clear orthologs of only two – *ASH1* and *WSC2* – were also identified as She-associated in *C. albican* ***s*** (Table 2 and [16]). Two possibilities could explain this difference: 1) the mRNAs transported by She3 differ considerably between the two species, or 2) the difference is an artifact of overly stringent filter criteria. To distinguish between these alternatives, we analyzed the *C. albicans* yeast form IP data to determine the percentile ranking of close homologs (including orthologs) of those mRNAs transported in *S. cerevisiae*. Excluding *ASH1* and *WSC2*, we identified clear *C. albicans* homologs (or, at least, best BLAST hits) for 13 of the *S. cerevisiae* She-transported mRNAs. We considered the percentile rank of all spots that met our basic threshold criteria (*i.e.*, produced interpretable hybridization in greater than 50% of arrays from one type of experiment); based on these criteria, array spots representing eleven genes were included in the analysis. The median percentile rank across all these array spots was 59, suggesting that these transcripts are not significantly enriched in the *C. albicans* She3-TAP IPs. When the same analysis was applied to the set of transcripts that were She3-associated in *C. albicans*, the median percentile rank was 99. Thus, the She machinery appears to bind distinct sets of transcripts in *C. albicans* and in *S. cerevisiae*.

The RNA elements that specify She3-dependent transport in *S. cerevisiae* are incompletely understood; they appear to be a complex combination of RNA secondary structure and RNA primary sequence [19],[39],[40],[41],[42]. For these reasons, simple sequence inspection of the transported mRNAs could not reveal whether the *C. albicans* She3-dependent transport system uses signals similar to those in *S. cerevisiae*.

### Localization of She3-associated transcripts

**Figure 2:**
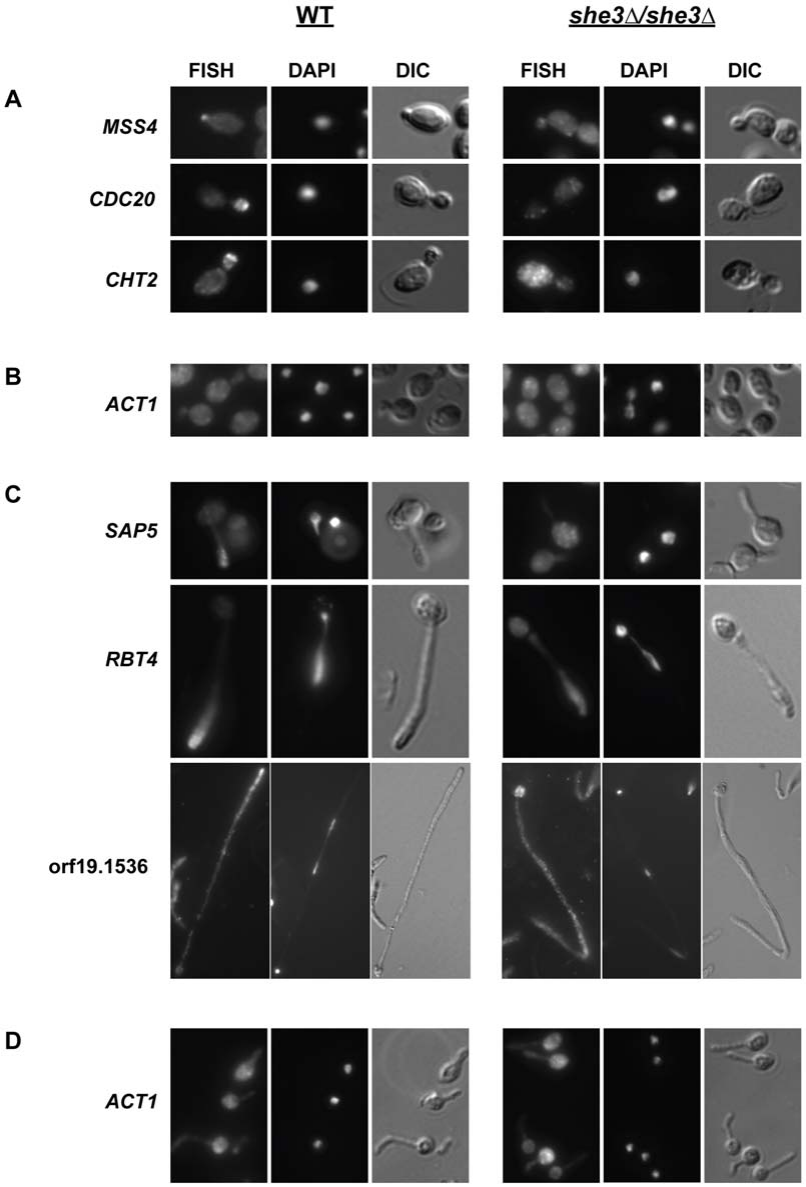
She3-associated transcripts accumulate in yeast buds and in hyphal tips. Cells from wild type (“WT,” CAF2-1) and *she3Δ/she3Δ* (SE4) strains were processed for FISH to detect endogenous She3-associated transcripts; cell nuclei were visualized with DAPI. Representative examples illustrate She3-dependent localization of the indicated transcripts. (A) Probe signal accumulates in the incipient bud (*MSS4*), or bud (*CDC20*, *CHT2*) of wild type *C. albicans* yeast. There is no specific localization of probe signal in yeast cells lacking She3. (B) A control *ACT1* probe is not localized in yeast cells from either strain. (C) In wild type hyphae, probe signal accumulates in the distal end of the germ tube (*SAP5*) or hyphal tip cell (*RBT4*, orf19.1536). Hyphae shown hybridized with *SAP5*, *RBT4*, and orf19.1536 probes were collected, respectively, 30 minutes, one hour, or three hours after serum induction. As in yeast, probe signals are not localized in hyphae lacking She3. (D) A control probe for *ACT1* is not localized in germ tubes from either strain collected 40 min after serum induction. Signals from two additional control probes, *ACC1* and *ADH1*, were not localized in yeast or hyphae (data not shown).

Based on these results, we predicted that transcripts bound to *C. albicans* She3 would accumulate in yeast daughter cells (buds) and in the tip cells of hyphae and that this accumulation would be She3-dependent. We tested this prediction for 21 transcripts bound by She3 using FISH. Each probe was hybridized to wild type and *she3Δ/she3Δ* cells under conditions in which the transcripts had been identified from the She3 IP experiments. Fourteen mRNAs were clearly detectable by FISH in the wild type background. In yeast cells, hybridization was observed in the presumptive bud site and/or the bud. In hyphae, signal accumulated at the distal tip of the germ tube (the nascent form of hyphae where a yeast cell sends out a long projection) and in the tip cells of mature hyphae. Signal accumulation in the bud and/or hyphal tip cell was absent in the *she3Δ/she3Δ* cells. Table 3 summarizes the results of the FISH experiments, and representative examples of She3-dependent RNA localization are shown in Figure 2; additional images are provided in Figure S2. In some cases, (*e.g.*, *CHT2* in yeast), fluorescence was clearly visible and diffuse in the mutant strain. In other examples *(e.g.*, *RBT4* in hyphae), the fluorescence in the mutant strain was not detectable above background. The lower signal in *she3Δ/she3Δ* cells, particularly in hyphae, suggests that site-specific accumulation is critical for visualizing the signal. It is unlikely that a lower signal in the deletion strain reflects reduced mRNA expression or stability; microarrays comparing the transcriptional profiles of *SHE3/SHE3* and *she3Δ/she3Δ* strains showed no overall decrease in levels of She3-associated transcripts (nine of the 14 probes with clear FISH results were analyzed; data not shown). In any case, the majority of probes (14/21) revealed that mRNAs identified as She3-bound were localized in a She3-dependent fashion, validating the IP and microarray analysis methods for identifying transported transcripts.

**Table 3:** Results of FISH experiments.

Probe		Cell Type			
		Yeast	Hyphae from Serum Induction		
			30 min	1 hr	3 hr
**Probes showing She-dependent localization**	CDC20	+++	+*		
	MSS4	++*	++		
	CHT2	+++	+*		
	PGA6			++	−−
	PGA55	++$	+	+	
	ASH1	+++#	+++#		+
	HAC1		++$		−−
	CCC1	−−	++		
	orf19.5406	−−	−−	++	
	orf19.1536		+		+++
	RBT4		++	+++	++
	SAP5		+		++
	orf19.4432		++		+++
	orf19.5224	−−	++		
**Probes with no detectable signal**	orf19.267	−−	−−	−−	
	orf19.5537		−−	−−	
	BCR1	−−	−−		
	ZCF11		−−	−−	−−
	orf19.1582		−−		
	CTA9	−−			
	YHB1		−−		

FISH experiments were performed in wild-type and *she3Δ/she3Δ* yeast and/or hyphae following 30 minutes, one hour, or three hours of serum induction, as indicated. A She3-dependent signal was detected for 14 probes in at least one cell type; for the remaining probes, no significant signal above background was detected.

+, ++, or +++ indicates probe enrichment in the yeast bud and/or hyphal tip in, respectively, <25%, 25–50%, or >50% of cells.

−− indicates absence of probe signal above background.

A blank box indicates that the probe was not tested in this cell type. Detection of certain probes was limited to certain stages of the cell cycle, as noted.

*: Pre-mitotic cells.

$ Cells with emerging buds or germ tubes.

# Post-mitotic cells.

In order to exclude the possibility that She3 transports all or most mRNAs in *C. albicans*, we performed FISH with three control probes, *ACT1* (orf19.5007), *ACC1* (orf19.7466) and *ADH1* (orf19.3997). These genes all had a median percentile rank of less than 75 in the She3 binding experiments. In each case, no specific localization was detected in yeast or in hyphae. Moreover, there was no detectable difference in distribution of the signal between wild type and *she3Δ/she3Δ* cells (Figure 2B and 2D). This result supports the conclusion that She3 localizes only a specific set of transcripts and that the *S. cerevisiae* and *C. albicans* She3 systems transport different mRNAs.

### She3 is necessary for invasive hyphal growth

**Figure 3:**
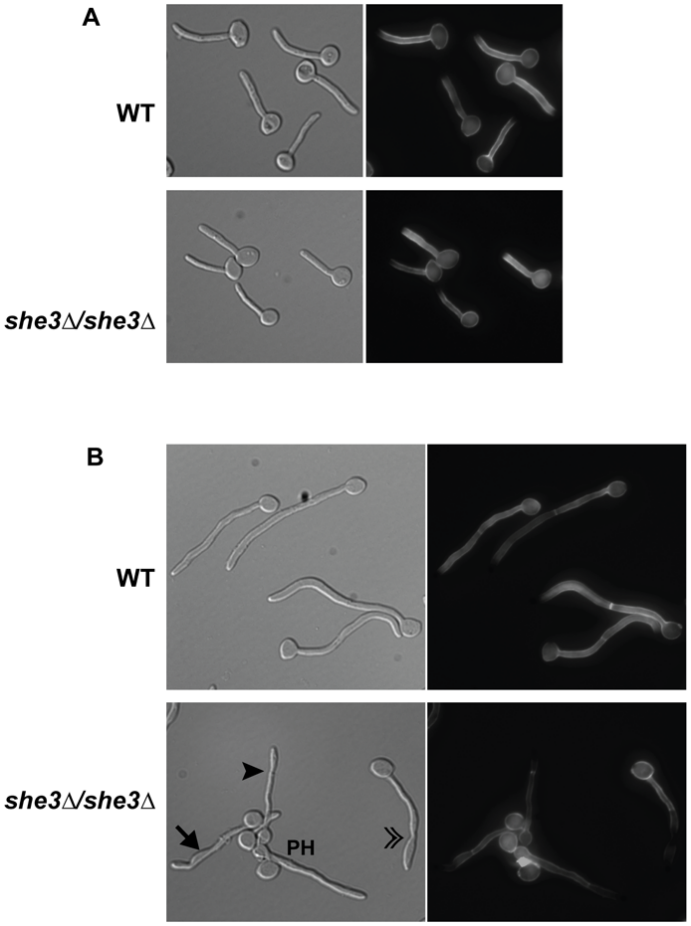
Morphology of *C. albicans* filaments from a *she3* null strain. Wild-type (“WT,” CAF2-1) and *she3Δ/she3Δ* (YSE4) strains were grown in YEPD/10% serum at 37°C for one (A) or two (B) hours, then fixed on cover slips and stained with Calcofluor White. (A) After one hour, germ tubes from the two strains are essentially indistinguishable. (B) By two hours, the majority of filaments from the *she3*-null strain display subtle defects, including swelling (→), uneven filament width (▸), and constrictions at the septal junctions in filaments that appear to have originally developed as true hyphae (»). The filament marked “PH” is a pseudohypha. The proportion of stereotypical and abnormal filaments from each strain at two hours after serum induction was determined. From 35–40 distinct fields, all fully visibly, un-branched hyphae with no constriction at junction of mother cell/filament junction were scored. Filaments displaying any of the above-described defects were scored as abnormal. Sixty-six percent of filaments from the *she3*-null strain (n = 167) showed some abnormality, whereas only five percent of wild type filaments (n = 171) displayed any defect.

Based on the role of She3 in localizing transcripts to the hyphal tip, we next investigated the requirement for She3 in the formation and proper function of hyphae. When grown in liquid serum-containing medium, the *she3Δ/she3Δ* strain forms germ tubes that are initially indistinguishable from those of the matched wild-type strain (Figure 3A), indicating that She3-regulated RNA transport is not required for the initiation of hyphal growth. However, subtle defects become apparent as the filaments grow. Normal hyphae have parallel sides with no constrictions at septal junctions, and their first septa are formed within the germ tube [28]. By two hours of serum exposure, approximately two-thirds of the *she3Δ/she3Δ* cells that had initiated germ tube formation failed to form normal hyphae; instead they displayed a range of defects, including constrictions at their septal junctions and uneven filament width (Figure 3B). By the same criteria, only five percent of wild type hyphae were abnormal. These data indicate that She3-mediated RNA transport is not required for germ tube formation, the earliest stage in hyphal formation, but comes into play at later stages of hyphal growth.

**Figure 4:**
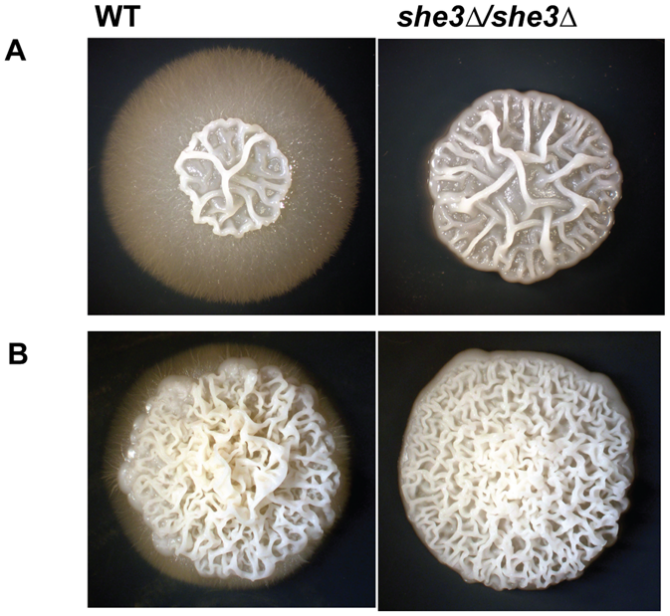
*C. albicans* lacking She3 are defective in invasive growth in agar. Wild type (“WT,” CAF2-1) and *she3Δ/she3Δ* (SE4) strains were grown for ten days on solid Spider medium (A) or on YEPD/10% serum medium (B). Images show representative colonies from each strain on each condition.

A more striking defect caused by deletion of the *SHE3* gene is observed on filament-inducing solid media (we use the term filament to include both hyphae and pseudohyphae). When grown on YEPD agar with 10% serum or on Spider agar, wild type *C. albicans* colonies develop a wrinkled central region (a mixture of yeast, hyphae and pseudohyphae), as well as peripheral filaments (predominantly hyphae) that invade the agar. *she3Δ/she3Δ* colonies specifically lack these peripheral filaments; the central wrinkled region is expanded, but otherwise indistinguishable from wild type colonies (Figure 4). This pronounced phenotype is observed at both 30°C and 37°C, and the identical defect was observed in six independently derived *she3Δ/she3Δ* strains, representing three different strain backgrounds. In the SN152 background [43], the *she3Δ/she3Δ* defect was complemented (that is, peripheral hyphae were restored) by re-introduction of the wild type *SHE3* gene (see Methods). While other loss-of-function mutations that preferentially affect central or peripheral filaments have been described [44], none of these completely and selectively eliminates peripheral filaments without affecting the central portion of the colony.

**Figure 5:**
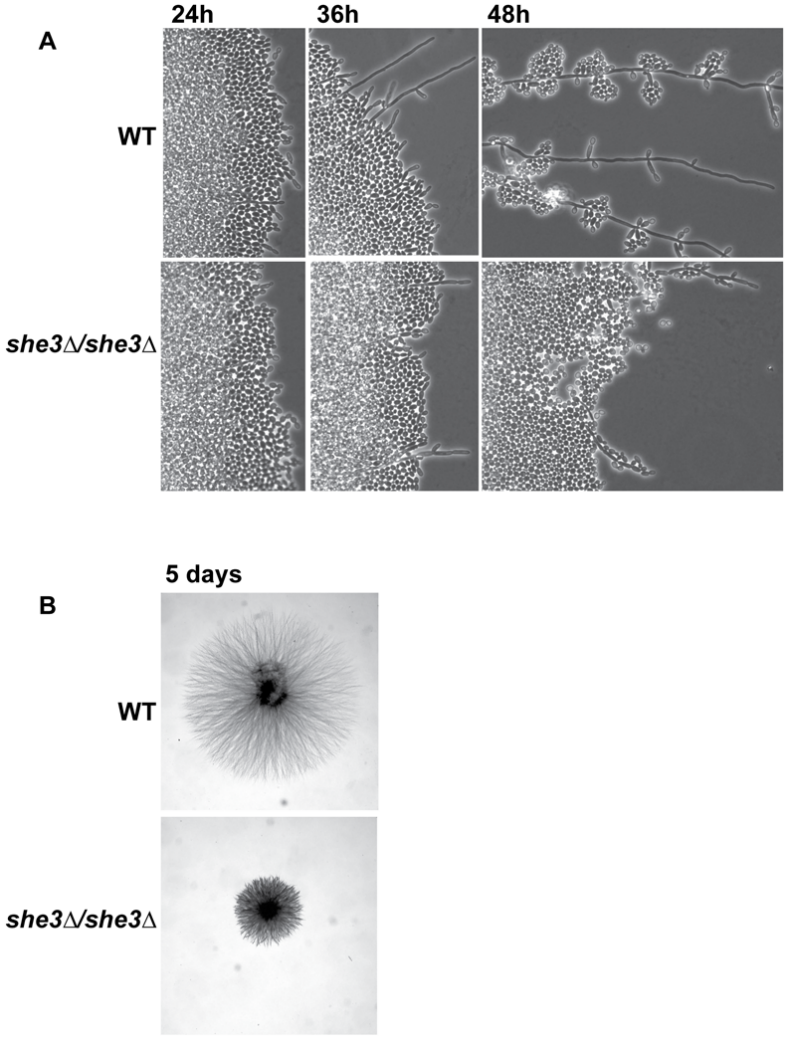
*C. albicans* She3 contributes to development of hyphae in solid media. (A) Colonies from wild type (“WT,” CAF2-1) and *she3Δ/she3Δ* (SE4) strains were grown on Spider agar slabs under a glass cover slip at 30°C. Images show typical colony edges at the indicated time periods. Similar results were observed on YEPD/10% serum slabs (data not shown). (B) Colonies from wild type and *she3Δ/she3Δ* strains grown for five days under a cover slip placed atop a Spider agar plate.

In order to visualize the defect caused by deletion of *SHE3* in greater detail, we monitored the initial events in the formation of peripheral filaments. Wild type and *she3Δ/she3Δ* cells were seeded onto thin agar slabs or standard agar plates and colony growth under a cover slip was observed for 48 hours. Colonies from both strains initially grew as yeast and began to filament by 36 hours. Early filaments of wild type colonies, observed at the interface between the agar and the cover slip, were a mixture of hyphae and pseudohyphae. Invasive filaments, which appeared by 48 hours, were predominantly hyphae. In the *she3Δ/she3Δ* strain, in contrast, hyphae were never observed (*i.e.*, all filaments were pseudohyphae), and the overall extent of filamentous growth and invasion of the agar was markedly decreased (Figure 5). These results, taken together, suggest that She3-regulated RNA transport is required for hyphal growth on solid media. It appears that the pseudohyphae of the *she3Δ/she3Δ* strain are inefficient at invasive growth, and that the absence of peripheral filaments in the *she3* null colonies stems from an inability to make invasive hyphae.

### She3 mediates epithelial cell damage

**Figure 6:**
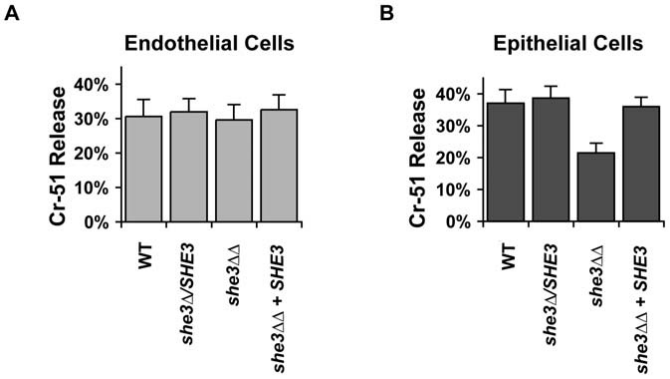
*C. albicans* lacking She3 shows reduced capacity to damage oral epithelial cells. Damage to human primary endothelial cells (A) or FaDu oral epithelial cells (B) induced by wild-type *C. albicans* (“WT,” QMY23), a *she3Δ/SHE3* heterozygote (SE67), a *she3Δ/she3Δ*homozygous deletion (SE63), or a *she3Δ/she3Δ* strain complemented with *SHE3* added to the *RPS1* locus (SE64). Results for endothelial and epithelial represent the mean +/− standard deviation of, respectively, two or three independent experiments.

We next tested whether the defect in invasive growth on solid agar might correlate with a defect in damage to host cells. A *she3Δ/she3Δ* strain was tested for the ability to damage monolayers of human epithelial and endothelial cells [45],[46]. While cells lacking *SHE3* were able to damage endothelial cells as efficiently as wild type, their ability to damage epithelial cells was reduced by about 40%, a statistically significant difference (Figure 6A). The defect suggests that tip-localization of one or more of the She3-associated transcripts may be required for the physical processes associated with epithelial cell damage or may be involved in sensing this particular niche. The *she3* null strain showed normal virulence in a mouse model of disseminated candidiasis (data not shown), suggesting that She3 is not required for this disease model [47].

Based on the sheer number of She3-transported mRNAs and the crucial functions predicted for some of the encoded proteins, one might have predicted that deletion of She3 would exhibit more severe phenotypes than those observed. It seems likely that She3 – mediated mRNA transport is one of several overlapping mechanisms to ensure proper protein localization. For example, the proteins encoded by She3-associated mRNAs could also contain localization signals (2,16). Alternatively, some of these proteins may retain all or part of their function even if mislocalized.

### Transported transcripts make diverse contributions to hyphal development

**Figure 7:**
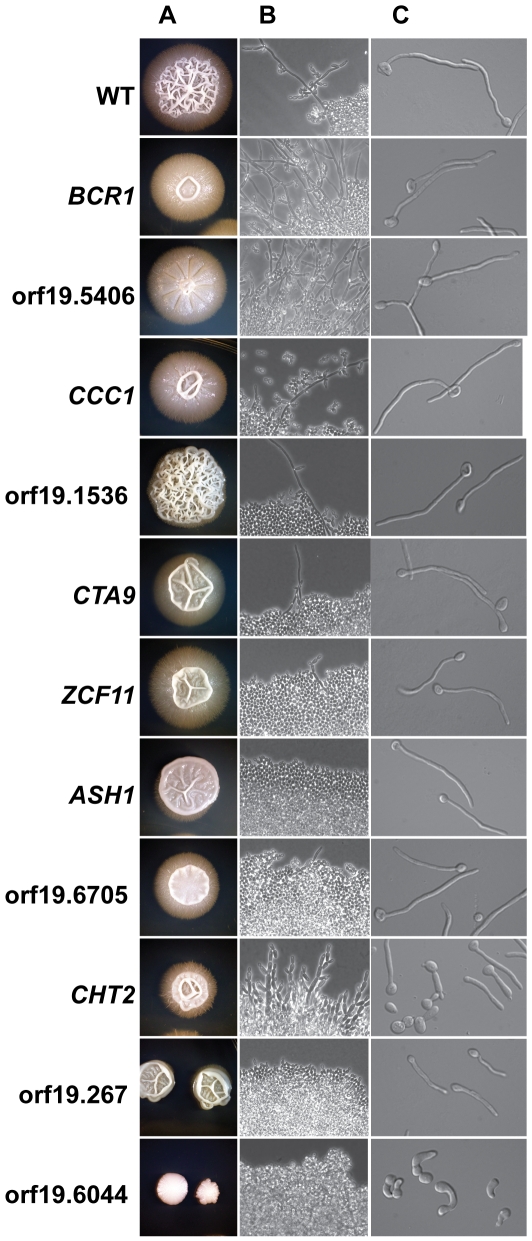
*C. albicans* strains lacking She3-associated transcripts are defective in filamentous growth. Wild-type *C. albicans* (“WT,” QMY23) or strains lacking the indicated transcripts were grown for ten days on solid Spider medium at 30°C (A), on Spider agar slabs under a glass cover slip at 30°C for 48 hours (B), or in liquid YEPD with 20% serum at 37°C (C). Images are representative of two isolates of each genotype.

To further explore the role of She3-mediated transport in hyphal development, we analyzed the roles of individual transported mRNAs. We constructed homozygous deletion strains for 33 of the genes encoding transported transcripts and assessed their phenotypes after ten days on Spider agar plates and after 48 hours on Spider agar slabs under a cover slip, as previously performed with the *she3Δ/she3Δ* strain. Eleven of the 33 mutants displayed colony morphology defects on Spider agar plates, and, among these, nine displayed aberrant filamentation in the early stages of embedded colony growth on Spider slabs. Some of the mutants showed an overall increase in filamentous growth, while some showed an overall decrease (Figure 7A and 7B). We tested the strains with aberrant colony morphology for the ability to form hyphae under strongly inducing conditions; *i.e.* exposure to serum at an elevated (37°C) temperature. Three strains, those lacking *CHT2*, orf19.6044, or orf19.267, displayed obvious defects. The phenotype of the orf19.6044 (*MOB2* ortholog) deletion mutant is consistent with Mob2's established role in polarized growth [48]. The remaining strains showed normal hyphal morphology in liquid serum-containing medium, suggesting that the deleted genes are required for specialized hyphal function but not for hyphal formation *per se*.

In summary, we analyzed deletion mutants corresponding to 33 transported mRNAs. Three strains exhibited severe defects in hyphal formation and an additional eight showed more subtle defects in hyphal growth regulation. These results support the conclusion that transport of specific mRNAs into the hyphal tip cell is needed for proper hyphal development and function.

## Discussion

In this paper, we describe an RNA transport system in *C. albicans* that localizes specific mRNAs to daughter cells in budding yeast and the tip cells of hyphae. When this RNA transport is inactivated by elimination of She3 (a component of the transport system), hyphae display specific defects, including aberrant growth and decreased capacity to damage an epithelial cell monolayer.

We identified mRNAs transported by this system through their tight association with She3, and we used FISH to show that the transported mRNAs accumulate in yeast buds and in the tips of hyphae in a She3-dependent manner. We believe that this study represents the first description of a set of mRNAs that are specifically localized to hyphal tip cells of a filamentous fungus.

Based on direct studies in *C. albicans* or characterization of orthologous genes in *S. cerevisiae*, the mRNAs bound by *C. albicans* She3 are predicted to encode several classes of proteins. Several (orf19.3356, *MSS4*, *CDC20*, orf19.267, orf19.3071, orf19.5537, *CHT2*, orf19.6044 and orf19.6705) encode proteins that function in mitosis, the cytoskeleton, cell wall dynamics, or cell polarity. Another group of associated mRNAs (*ASH1*, *CTA9*, *CTA9*, *BCR1*, *HAC1*, *GLN3*) encode transcriptional regulators. She3 also associates with mRNAs for cell-surface proteins, including predicted GPI anchored proteins (*PGA55*, *YWP1*, *PGA6*, and *PGA54*) and cell membrane-associated regulators of ion transport (orf19.1582 and orf19.5406). Finally, She3-associated RNAs encode known hyphal-specific virulence factors, *RBT4* and *SAP5*. Taken together, the identities of transported mRNAs suggest that the She3 machinery supports diverse functions, including localization of the basic machinery required for cellular growth and polarity, specification of transcriptional programs in daughter cells and in hyphal tip cells, and differential distribution of cell surface and secreted proteins, some of which function in virulence.

We present several lines of evidence that She3-mediated RNA transport, although not required for hyphal formation per se, is required for normal hyphal growth and function. First, *she3Δ/she3Δ* strains display reduced ability form hyphae and to penetrate solid agar. Second, although *she3Δ/she3Δ* strains can form hyphal structures in certain conditions, these filaments are morphologically abnormal. Third, a *she3Δ/she3Δ* strain shows reduced capacity to damage an epithelial cell monolayer. Finally, we constructed and tested deletions of 33 genes whose transcripts are She3-bound. Approximately one third of the individual deletion mutants have filamentation defects on solid medium, and the aberrant morphologies vary considerably among the mutants. As might be expected, none of these strains displayed exactly the same defects as the *she3Δ/she3Δ* strains, indicating that the *she3* mutant phenotype does not reflect the absence of a single transported mRNA in hyphal tip cells. Taken together, these observations support the idea that transport of multiple mRNAs to hyphal tip cells contributes to proper hyphal function.

Our analysis of the She system in *C. albicans* allows for the first direct cross-species comparison of an RNA transport system. A surprising finding from our studies is the minimal apparent overlap between She-associated transcripts in *S. cerevisiae* and *C. albicans*: only two genes (out of 40 in *C. albicans* and 24 in *S. cerevisiae*) are bound in both species. These results suggest that specific mRNAs have moved in and out of the She3-dependent transport system relatively rapidly over evolutionary timescales.

Based on existing data, two plausible mechanisms could account for the apparently rapid evolution of mRNAs transported by the She system. In one model, changes in the RNA-binding specificity of the modular She complex could account for this difference. In an alternate model, which we favor, the change in She3 cargo may have arisen via changes in the nucleotide sequences of the transported mRNAs, which have brought new transcripts under She3 regulation. The *cis*-acting elements that mediate localization of She-associated transcripts in *S. cerevisiae* have been characterized for a small subset of transported RNAs and are composed of short degenerate sequence motifs, as well as secondary structures that are largely sequence-independent [19],[39],[40],[41],[42]. It is plausible that, over evolutionary timescales, small sequence changes mediate rapid losses and gains of cargo mRNAs. Such a mechanism is analogous to evolutionary changes in transcription circuitry, where the basic transcriptional machinery and its regulators have been conserved over long timescales, but changes in cis-regulatory sequences have brought new sets of genes in and out of control of ancient regulators [49],[50].

*C. albicans* and *S. cerevisiae* diverged from a common ancestor roughly 200 million years ago, and since that time they have adapted to distinct environmental niches. *S. cerevisiae* is widely distributed in the environment, whereas *C. albicans* is restricted to warm-blooded animals. We suggest that the differences in the She3-transported mRNA cargos likely reflect the distinct pressures of each organism's environmental niche.

## Materials and Methods

### Strains and media

Strains used in this study are listed in Table 1 and described in greater detail below and in Text S1. CAI4, CAF2-1, SN87, SN152, and QMY23 have been described previously [43],[51],[52]. *C. albicans* transformations were performed according to standard lithium acetate methods. For cultivation of *C. albicans* hyphae, strains were grown to OD 10–12, then diluted at least tenfold into YEPD containing 10% serum and grown at 37°C, unless otherwise indicated.

### Strain construction

Two methods were used for deleting orf19.5595 (*SHE3*). A modified Ura-blaster protocol [53] was used for construction of the heterozygous deletion strain SE6 and homozygous deletion strains SE4 and SE5. Fusion PCR methods that avoided using the *URA3* marker were used to produce the *she3* null mutants SE30 and SE32, in, respectively, SN87 and SN152 backgrounds [43]. For complementation studies, a construct containing *SHE3* under the control of its own regulatory sequence was introduced to the region downstream of RPS1 (orf19.3002) in SE32 to generate the *SHE3*-complemented strain SE64. *she3* heterozygote and null mutant strains with the same nutritional markers as SE64 were also generated.

Strains SE18 and SE20 in which one copy of *ASH1* contained an amino-terminal 6×MYC tag were generated in, respectively, wild type (CAI4) and *she3-null* backgrounds. One copy of endogenous *ASH1* was deleted, and the plasmid p DI-30 [35], carrying 6MYC-ASH1, was integrated into the region of the deleted ASH1 allele.

A strain in which the single copy of *SHE3* was TAP-tagged [37] was constructed using modified fusion PCR methods [43] to produce the *SHE3-TAP* strain (SE25), in which a TAP-URA3 cassette was added immediately upstream of the stop codon of the *SHE3* allele.

Deletion strains corresponding to individual She-associated transcripts were constructed in the SN152 background using fusion PCR methods [43].

Detailed methods of strain construction are provided in Text S1; primers used in strain construction are provided in Table S1.

### Immunoprecipitation of She3-RNA complexes and microarray analysis

Immunoprecipitation of She3-RNA complexes from SE25 were adapted from published methods [16],[20] and are described fully in Text S1. Briefly, exponentially growing yeast cells or hyphae produced by 30 minutes, one hour, or three hours of serum induction were lysed with glass beads in extraction buffer. Lysates were incubated with IgG-sepharose beads, the immunoprecipitate was released from the beads by cleavage with TEVprotease, and RNA was isolated by phenol-choloroform extraction followed by ethanol precipitation. For a mock RNA immunoprecipitation, the SHE3-TAP parental strain SE6 was subjected to the same methods. Total RNA from the SHE3-TAP strain was harvested from cultures prepared as above and was isolated by a hot phenol protocol [54]. Labeled cDNA was generated from each RNA sample, coupled to fluorescent dyes and hybridized to DNA microarrays essentially as described [38]. Microarray data were quantified using GENEPIX PRO version 3.0 or 5.0 and were further processed using NOMAD (http://ucsf-nomad.sourceforge.net/). Processed data were analyzed in Microsoft Excel; filters applied to the data are described in the Results. cDNA samples from generated from total RNA from SE25 and SE6 were directly compared on DNA microarrays using the above methods.

### Fluorescent *in situ* hybridization (FISH)

Methods were adapted from published protocols [55] and are described in detail in Text S1. Briefly, for each FISH probe, a digoxigenin-labeled antisense riboprobe was generated by *in vitro* transcription from a plasmid template containing a portion of the corresponding gene; primers used for template construction are provided in Table S2. Yeast and hyphal cells were grown as described above, fixed in 5% formaldehyde, and spheroplasted in sorbitol buffer containing zymolyase 100 T. Probe hybridization and signal detection with the HNPP Fluorescent Detection Set (Roche) were performed essentially as described [24]. Mounted cells were imaged on the Axiovert-200 (Carl Zeiss, Thornwood, NY).

### Preparation of hyphae on coverslips

*C. albicans* strains were grown to approximately OD-12 in YEPD at 30°, diluted 1∶50 into YEPD/20% serum, and incubated with rotation for two hours at 37°C. Cultures were fixed in culture medium plus 4% formaldehyde for one hour, washed and resuspended in PBS, and sonicated in Branson Sonifier 450 for 30 seconds with power setting 1.5, 40% duty cycle. Cells were adhered for ten minutes to cover slips that had been pretreated overnight with 1 mg/ml concanavalin A, washed twice in PBS, then stained with 1 µg/ml fresh Calcofluor White for ten minutes in the dark. Cover slips were washed five times with PBS, and then mounted on glass slides.

### Preparation of colonies on agar slabs

Agar slabs were prepared by pouring molten agar media between glass plates separated by 1 mm spacers; rectangular pieces of the solidified media were places atop glass slides. The slabs were spread with 10 µl of *C. albicans* preparations (exponentially growing *C. albicans* yeast cultures, diluted to approximately 1 cfu/µl in water), overlaid with glass coverslips, and kept in a humid chamber at 30°C.

### Cell damage assays

Endothelial and epithelial cell damage assays were performed as previously described [45],[46].

## Supporting Information

Figure S1 Schematic representation of the methods used to identify She3-associated transcripts. A TAP-tagged version of She3 was immunoprecipitated from lysates from *C. albicans* yeast and from hyphae collected 30 minutes, one hour, or three hours after serum induction. The associated mRNAs (it is not clear whether She3 binds RNA directly or indirectly) were eluted and used to generate cDNA for microarray analysis. Fluorescently labeled cDNA from She3-associated transcripts was competitively hybridized against reference cDNA derived either from total RNA from the She3-TAP strain or from a mock IP with the parental strain (derived from four pooled mock IPs). Twelve microarrays from yeast (6 each using either the two reference samples) and 24 from hyphae (from each of three time points, 4 four arrays each using the two reference populations) were performed to determine the set of She3-associated RNAs.
(0.08 MB PDF)

Figure S2 She3-associated transcripts accumulate in yeast buds and in hyphal tips; images not shown in Figure 2. Cells from wild type (“WT,” CAF2-1) and *she3Δ/she3Δ* (SE4) strains were processed for fluorescent in situ hybridization (FISH) to detect endogenous She3-associated transcripts; cell nuclei were visualized with DAPI. (A) PGA55 probe signal accumulates in the bud of wild type *C. albicans* yeast. There is no specific signal in *she3Δ/she3Δ* yeast cells (data not shown). In wild-type hyphae collected 30 minutes (B), one hour (C), or three hours (D) after serum induction, the probe signal accumulates in the distal end of the germ tube or hyphal tip cell. There is no specific localization in hyphae lacking She3. Probe identities are as indicated.
(3.59 MB PDF)

Table S1 Primers used for strain construction, as described in the Supporting Materials and Methods section (Text S1).
(0.04 MB PDF)

Table S2 Primers used for generation of FISH probes. The Description column lists gene name (as in Table 2) and primer orientation. The reverse primers include T7 promoter sequence, which is in lowercase.
(0.05 MB PDF)

Table S3 Fold enrichment of transcripts identified as She3-associated in microarray experiments comparing transcripts immunoprecipitated with She3 to reference samples. For each She3-associated transcript, raw enrichment values (ratio of the medians of She3-associated RNA compared to reference) are provided for each experiment (*i.e.*, one growth condition using one reference population) where array element(s) representing that transcript passed the initial filter - spots produced interpretable hybridization in greater than 50% of arrays from that experiment and had a median percentile rank of at least 98. In cases where individual transcripts are represented by multiple array elements, the highest enrichment value from each microarray is provided. Microarrays #1–12 represent experiments with yeast cells. Microarrays #13–20, 21–28, and 29–36 represent experiments with hyphae grown in serum for, respectively, 30 minutes, one hour, or three hours. For microarrays # 1–6, 13–16, 21–24, and 29–32, the reference was RNA from a mock immunoprecipitation, as described in the Results. For microarrays # 7–12, 17–20, 25–28, and 33–36, the reference sample was total RNA from the She3-TAP strain.
(0.04 MB XLS)

Table S4 Raw microarray data. For the 36 arrays used to compare She3-associated and reference mRNA, raw enrichment values (ratio of the medians) are provided for all array elements (spots) that produced interpretable hybridization. “Element ID” refers to the unique identifier assigned to each array spot. “Candida albicans ORF” refers to the gene identifier associated with the sequence of that spot, as determined at the time the microarrays were produced. Microarrays #1–12 represent experiments with yeast cells. Microarrays #13–20, 21–28, and 29–36 represent experiments with hyphae grown in serum for, respectively, 30 minutes, one hour, or three hours. For microarrays # 1–6, 13–16, 21–24, and 29–32, the reference was RNA from a mock immunoprecipitation, as described in the manuscript Results. For microarrays # 7–12, 17–20, 25–28, and 33–36, the reference sample was total RNA from the She3-TAP strain.
(2.36 MB XLS)

Text S1 Supporting materials and methods.
(0.08 MB PDF)
