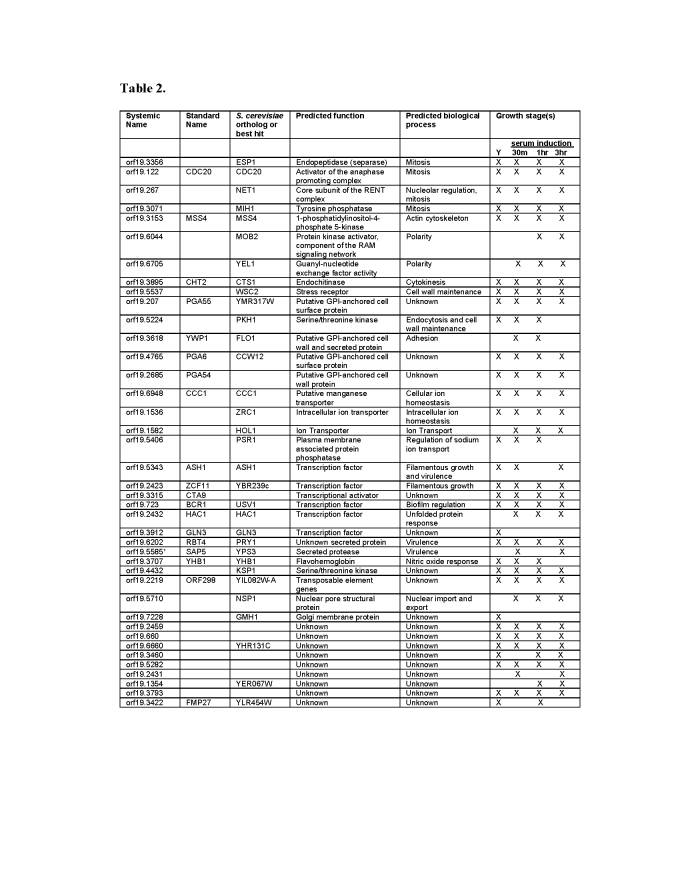# Correction: An RNA Transport System in *Candida albicans* Regulates Hyphal Morphology and Invasive Growth

**DOI:** 10.1371/annotation/17eb3a67-8f49-454b-acbf-5ff2872c27ff

**Published:** 2009-10-19

**Authors:** Sarah L. Elson, Suzanne M. Noble, Norma V. Solis, Scott G. Filler, Alexander D. Johnson

Table 2 was not formatted correctly in the published article. The correct version is available here: 

**Figure:**