# Supplementary figures and images for: An RNA Transport System in Candida albicans Regulates Hyphal Morphology and Invasive Growth
Source: PLoS Genet. 2009 Sep 25;5(9):e1000664. doi: 10.1371/journal.pgen.1000664 (PMC2739428; doi:10.1371/journal.pgen.1000664)

Supporting Figure S1.

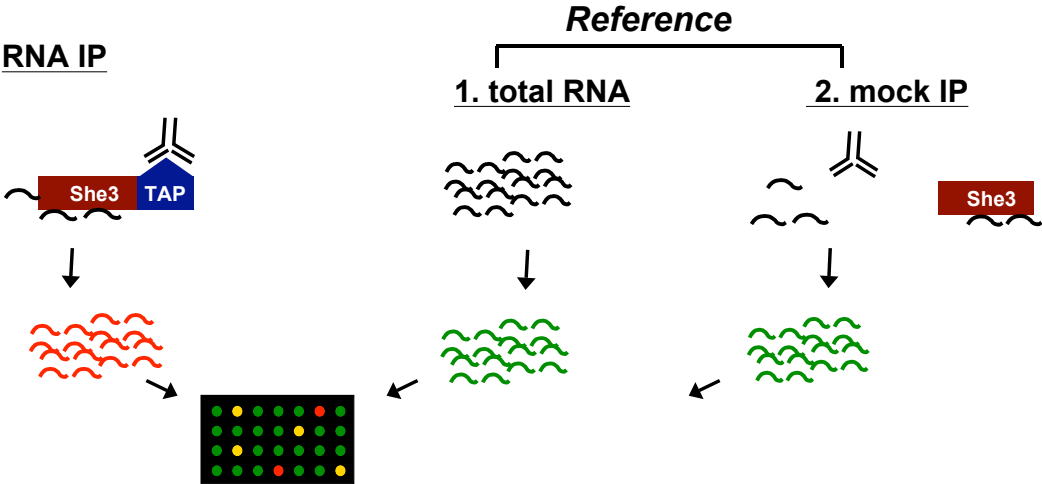

Supplement: Figure S1 — Schematic representation of the methods used to identify She3-associated transcripts. A TAP-tagged version of She3 was immunoprecipitated from lysates from C. albicans yeast and from hyphae collected 30 minutes, one hour, or three hours after serum induction. The associated mRNAs (it is not clear whether She3 binds RNA directly or indirectly) were eluted and used to generate cDNA for microarray analysis. Fluorescently labeled cDNA from She3-associated transcripts was competitively hybridized against reference cDNA derived either from total RNA from the She3-TAP strain or from a mock IP with the parental strain (derived from four pooled mock IPs). Twelve microarrays from yeast (6 each using either the two reference samples) and 24 from hyphae (from each of three time points, 4 four arrays each using the two reference populations) were performed to determine the set of She3-associated RNAs. (0.08 MB PDF) [file pgen.1000664.s001.pdf]

Supporting Figure S2

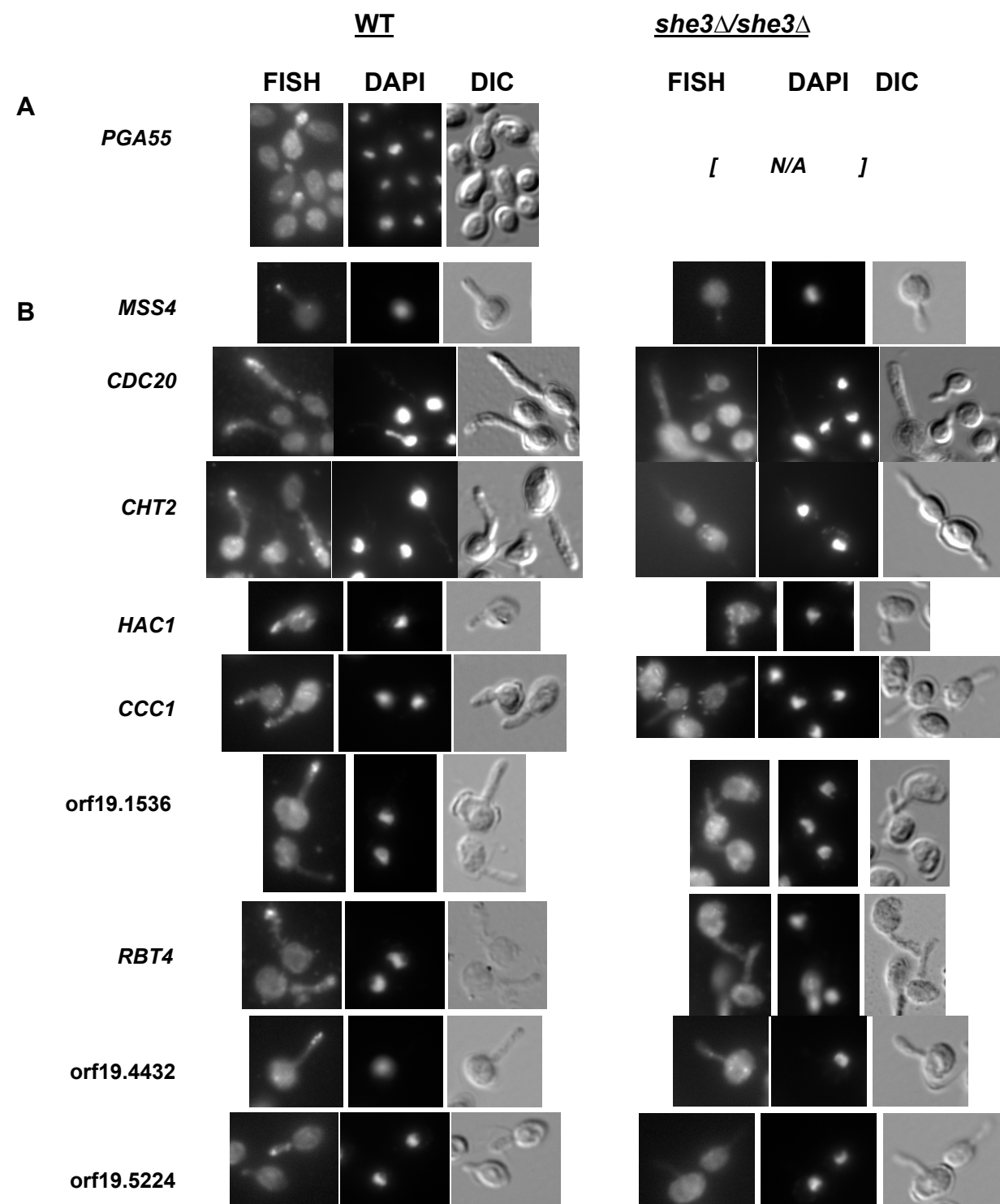

Supporting Figure S2

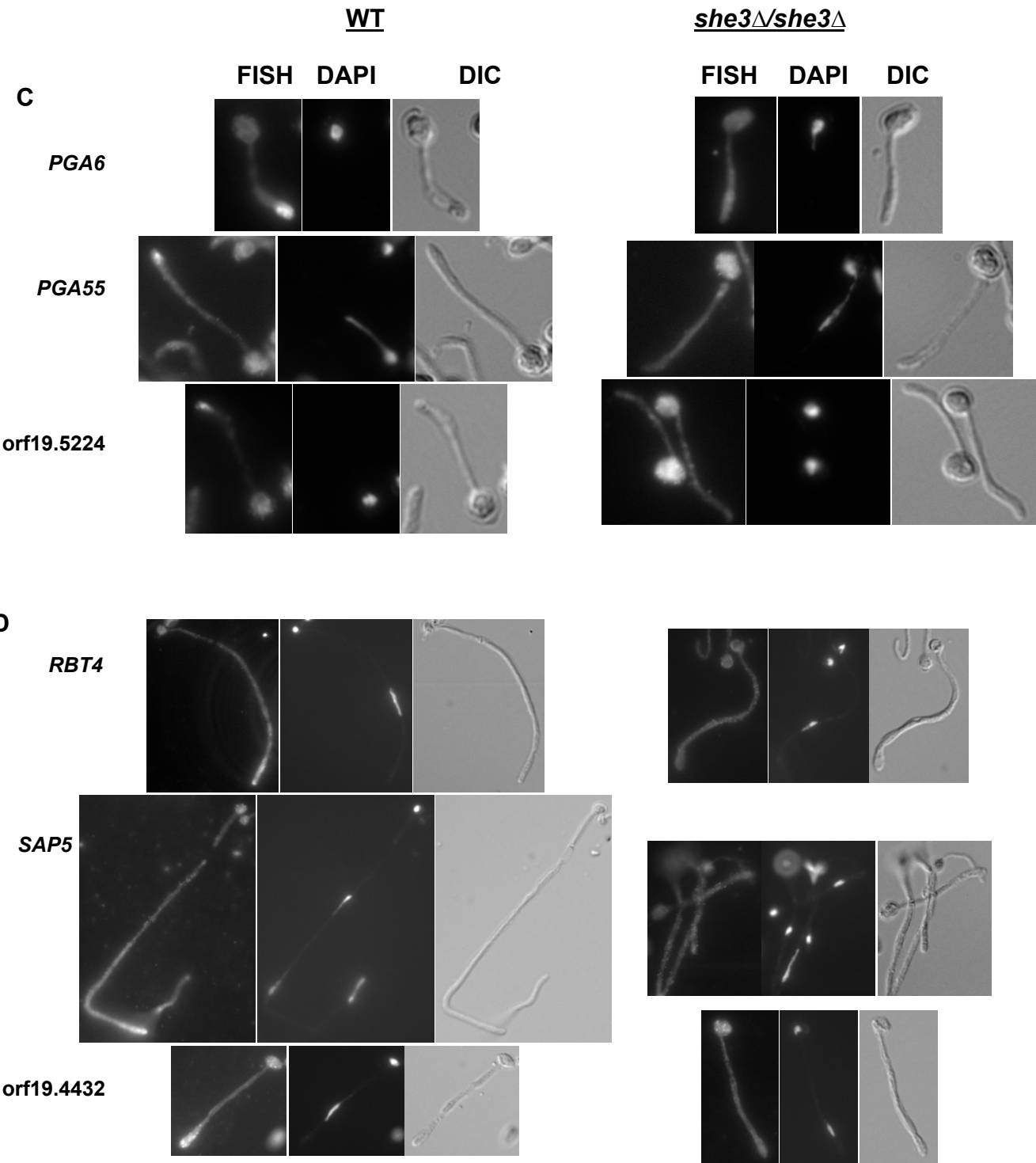

Supplement: Figure S2 — She3-associated transcripts accumulate in yeast buds and in hyphal tips; images not shown in Figure 2. Cells from wild type (“WT,” CAF2-1) and she3Δ/she3Δ (SE4) strains were processed for fluorescent in situ hybridization (FISH) to detect endogenous She3-associated transcripts; cell nuclei were visualized with DAPI. (A) PGA55 probe signal accumulates in the bud of wild type C. albicans yeast. There is no specific signal in she3Δ/she3Δ yeast cells (data not shown). In wild-type hyphae collected 30 minutes (B), one hour (C), or three hours (D) after serum induction, the probe signal accumulates in the distal end of the germ tube or hyphal tip cell. There is no specific localization in hyphae lacking She3. Probe identities are as indicated. (3.59 MB PDF) [file pgen.1000664.s002.pdf]
