# Supplementary material for: An RNA Transport System in Candida albicans Regulates Hyphal Morphology and Invasive Growth
Source: PLoS Genet. 2009 Sep 25;5(9):e1000664. doi: 10.1371/journal.pgen.1000664 (PMC2739428; doi:10.1371/journal.pgen.1000664)
Supplement: Table S1 — Primers used for strain construction, as described in the Supporting Materials and Methods section (Text S1). (0.04 MB PDF) [file pgen.1000664.s003.pdf]

Supporting Table S1.

Primers used in strain construction

Primer name	Sequence
SEO1	CACCCCAACCAGTTCCAATATTCATTTAATTTTTGATTGATTGAATATTA ACAAGTAGAGATGTTTTCCCAGTCACGACGTT
SEO2	GCAGGATATGAAGATGGTTCAATACTTGCCTACAATATATAGTTAATTCC TTCATCGTTATCCTGTGGAATTGTGAGCGGATA
SEO3	CCAGTTCCAATATTCATTTAATTTTTGATTGATTGAATATTAACAAGTAGAGATACACACCCAGATCAAC ACCCTCATAGGTTTTCCCAGTCACGACGTT
SEO4	TCAATACTTGCCTACAATATATAGTTAATTCCTTCATCGTTATCCATTTTTTTAAAAAAAAACTAACGTGT AATATGCTCTGTGGAATTGTGAGCGGATA
SEO14	CACCGTCGTCAACCAAGTCAGC
SEO18	GCAGGGATGCGGCCGCTGACGAATTCGAGCTCGTTTAAAC
SEO19	GTCAGCGGCCGCATCCCTGCTAGTATAGAGGTTTATATTTTTAGAGC
SEO20	GGCAACACTGAACAAGAAAAATCG
SEO27	GGCTATGAAATTCTTTTTCCATCTTCTCTTTTCCATTACATTTAATGCAAAGTTATTATCG
SEO28	ATGGAAAAGAGAAGATGGAAAAAGAATTTCATAGCC
SEO112	GTTCTTCCCCCAAAACATTAG
SEO113	CACGGCGCGCCTAGCAGCGGGTGTTGTTATTTTCTTTTAAACG
SEO120	TAGATGGATCCCTCGACTATTACTCATTGATAAAGAC
SEO121	CTGTACTCTAGAGGATAATGATAACTGAAGAGAAG
SEO126	ACCTGTACGCGGCCGCCCATTGCATGTATCAGCAAGG
SEO127	AGGCTCTCGAGTTAATTCCTTCATCGTTATCC
