# Supplementary material for: An RNA Transport System in Candida albicans Regulates Hyphal Morphology and Invasive Growth
Source: PLoS Genet. 2009 Sep 25;5(9):e1000664. doi: 10.1371/journal.pgen.1000664 (PMC2739428; doi:10.1371/journal.pgen.1000664)
Supplement: Table S2 — Primers used for generation of FISH probes. The Description column lists gene name (as in Table 2) and primer orientation. The reverse primers include T7 promoter sequence, which is in lowercase. (0.05 MB PDF) [file pgen.1000664.s004.pdf]

Supporting Table S2.

Primers used for generation of FISH probes

Primer name	Sequence	Descripton
DIO8	TCAAGACAAATCACAATTCC	ASH1-for
DIO116	taatacgactcactatagTTGTTGATTATTCGGTATAGAG	ASH1-rev-T7
SEO68	GTCTCTTGTTGCAATAAAAAACGG	CCC1-for
SEO69	GTtaatacgactcactatagggagaAGAACACCAATCCCCAAGCG	CCC1-rev-T7
SEO70	GATTCAATTGATCCATCGTCC	CDC20-for
SEO71	GTtaatacgactcactatagggagaCTTTTACTATCATGACCACCG	CDC20-rev-T7
SEO72	TCATCAGCTTTGGCCAGTGCC	CHT2-for
SEO73	GTtaatacgactcactatagggagaAGCCAATAAGAATGGAACAGG	CHT2-rev-T7
SEO76	CATTATCAAGTGAGTCCTGCC	PGA55-for
SEO77	GTtaatacgactcactatagggagaTGTCTTGATGCTGCGTTTCCC	PGA55-rev-T7
SEO78	ATGCCTATGCCTATGCTACCG	MSS4-for
SEO79	GTtaatacgactcactatagggagaCATATTCTCTAGCAGGAACCG	MSS4-rev-T7
SEO80	TATCTTGTGGACCGTACAGCG	orf19.267-for
SEO81	GTtaatacgactcactatagggagaAAGGTGACAGTGGGTCACTCG	orf19.267-rev-T7
SEO82	TGACAAATCCAAAGACAGTCC	orf19.5224-for
SEO83	GTtaatacgactcactatagggagaGAGCAATAACTGGACCAATACG	orf19.5224-rev-T7
SEO84	CGATGACAAAGTACACCGACC	orf19.5406-for
SEO85	GTtaatacgactcactatagggagaCATCATCAACAATTGGCTTTGC	orf19.5406-rev-T7
SEO86	GACCTGTGACTCGGGTCGTCC	ZCF11-for
SEO87	GTtaatacgactcactatagggagaCACAATCATCATGGGAATACC	ZCF11-rev-T7
SEO88	ATGCAATTCCAAACTTTATTAGTCG	PGA6-for
SEO89	GTtaatacgactcactatagggagaAAGATAGCGGCTAAACCAGC	PGA6-rev-T7
SEO90	GATTACCTTAAATTCCCTGGC	CTA9-for
SEO91	GTtaatacgactcactatagggagaCTAGCAATTCTACTGCTGGG	CTA9-rev-T7
SEO92	GAGTTAACTGTTGATAACACC	HAC1-for
SEO93	GTtaatacgactcactatagggagaATTCTATTGGCTCTGCTGGC	HAC1-rev-T7
SEO94	GTTTTTTGTATTGGAAGATACAAC	orf19.1582-for
SEO95	GTtaatacgactcactatagggagaCTTTCATATGGTTCAGCAGTCC	orf19.1582-rev-T7
SEO98	CGCGTAACAATGACTGTTTGG	orf19.4432-for
SEO99	GTtaatacgactcactatagggagaCAATTTGGCTCATTGTACTTCC	orf19.4432-rev-T7
SEO100	TTTGCTGGTTTAACCACTGC	RBT4-for
SEO101	GTtaatacgactcactatagggagaGGTCTCAAGACATTTTCAGC	RBT4-rev-T7
SEO102	GCTTTATTAATTGATGCTGCTCC	SAP5-for
SEO103	GTtaatacgactcactatagggagaGAATATTATCTTCACTTTCACG	SAP5-rev-T7
SEO104	TCCCATCAATCACAGCACGC	BCR1-for
SEO105	GTtaatacgactcactatagggagaCGTACCACCACCTGTTATGC	BCR1-rev-T7
SEO106	CAGTATATGCTGACAGTTGG	orf19.5537-for
SEO107	GTtaatacgactcactatagggagaCAAAGATCCAGGATTTCGAGC	orf19.5537-rev-T7
SEO108	TGACAGTCGAATACGAAACC	YHB1-for
SEO109	GTtaatacgactcactatagggagaCAGGAACATTCTTTTTACCC	YHB1-rev-T7
SEO110	GGGTATACTGTCCATTCATTAGC	orf19.1536-for
SEO111	GTtaatacgactcactatagggagaTAGAAAATCAGACGACACAC	orf19.1536-rev-T7
