# Supplementary material for: An RNA Transport System in Candida albicans Regulates Hyphal Morphology and Invasive Growth
Source: PLoS Genet. 2009 Sep 25;5(9):e1000664. doi: 10.1371/journal.pgen.1000664 (PMC2739428; doi:10.1371/journal.pgen.1000664)
Supplement: Text S1 — Supporting materials and methods. (0.08 MB PDF) [file pgen.1000664.s007.pdf]

SUPPORTING MATERIALS AND METHODS

Strain construction

Supporting Table S1 lists primers used for strain construction in this study. *SHE3* was deleted using a modified Ura-blaster protocol [1]. The recyclable *URA3* marker was PCR-amplified from pDDB57 using long oligonucleotides identical to the sequence flanking the *SHE3* ORF (SEO1 and SEO2). The disruption fragment was transformed into the CAI4 [2] strain. The resulting heterozygote was plated onto 5-fluoro-orotic acid (5-FOA) and uridine to select for loss of *URA3*. A second round of transformation, which used primers SEO3 and SEO4 to amplify *URA3*, generated the *she3*-null strain SE4. SE4 was again subjected to 5-FOA selection to yield the URA- *she3*-null strain SE5.

SHE3 was also deleted using fusion PCR methods, as described, that avoided using the *URA3* marker [3]. *SHE3* 5' and 3' flanking sequences were PCR-amplified with primer pairs SEO112 / SEO113 and SEO19/SEO20, respectively, and used to generate *HIS1* and *LEU2* disruption fragments. Strains SN87 and SN152 were transformed with the *she3::HIS1* fragment, and the resulting heterozygotes (SE28 is the *she3::HIS/SHE* derivative of SN152) were transformed with *she3::LEU2* to produce the null mutants SE30 and SE32, in, respectively, SN87 and SN152 backgrounds.

For complementation studies, a construct containing *SHE3* under the control of its own regulatory sequence was introduced to the region downstream of *RPS1* (orf19.3002) in the *she3* null mutant SE32. The plasmid pSN67, containing the *ARG4* marker cloned into pCR-BluntII (Invitrogen) is the same as pSN69 [3] except that *ARG4* is in the opposite orientation. The *RPS1* downstream region was amplified with primers SEO120

and SEO121 and cloned into BamHI and SpeI sites in pSN67 to generate pARG4r. A SHE3 fragment was amplified with primers SEO126 and SEO127, cut with NotI on the 5' end (the 3' end was left blunt) and cloned into Not1 and blunted XhoI sites of pARG4r to generate pSHE3-ARG4r. pSHE3-ARG4r was integrated into the NruI site downstream of RPS1 in SE32 to generate SE64. Testing on Spider-agar medium [4] confirmed that the introduced *SHE3* gene complemented the filamentation defect (see Results). *she3* heterozygote and null mutant strains with the same nutritional markers as SE64 were also generated. pARG4r was linearized with NruI and transformed into SE28 and SE32 to generate SE61 and SE63, respectively. SE61 was additionally transformed with a *LEU2* fragment at the site of the deleted endogenous *LEU2* gene (as in QMY23 [5]) to generate SE67.

Strains in which one copy of *ASH1* contained an amino-terminal 6xMYC tag were generated in wild type (CAI4) and *she3-null* backgrounds. An *ASH1* disruption fragment was excised from pDI-03 [6] with HindII and KpnI and transformed into CAI4 and SE5 to generate, respectively, *ash1::URA3/ASH1* and *ash1::URA3/ASH1*; *she3Δ/she3Δ*. Both strains were plated on 5-FOA and uridine to select URA- derivatives. The plasmid pDI-30 [6], carrying 6*MYC-ASH1*, was integrated at the Pac1 sites in the region upstream of the deleted *ASH1* alleles of these derivative strains to yield *ash1::p6MYC-ASH1::URA3/ASH1* (SE18) and *ash1::p6MYC-ASH1::URA3/ASH1*; *she3Δ/she3Δ* (SE20). Expression of the *6MYC-ASH1* gene was confirmed with western blotting.

A strain in which the single copy of *SHE3* was TAP-tagged [7] was constructed. A plasmid containing a TAP-*URA3* cassette was generated by amplifying *URA3* from pDDB57 [1] and cloning it into AscI and PmeI sites of the pFA6a-TAP plasmid from

which the HIS3MX cassette had been excised [8]. Modified fusion PCR methods [3] were used to generate a tagging cassette comprising the 3' end of the *SHE3* coding sequence, amplified with primers SEO14 and SEO27, the TAP-*URA3* cassette, amplified with primers SEO28 and SEO18, and the *SHE3* stop codon and downstream region, amplified with primers SEO19 and SEO20. SE6 was transformed with the tagging cassette to generate the *SHE3-TAP* strain (SE25). Expression of the *SHE3-TAP* gene was confirmed with western blotting using an antibody to the calmodulin binding peptide portion of the TAP tag (Upstate #07-428), and functionality of She3 was confirmed by observation of peripheral filaments when the strain was grown on Spider agar (see Results).

Immunoprecipitation of She3-RNA complexes and microarray analysis

Approximately 250 OD units of exponentially growing yeast cells, or an equivalent volume of hyphal cells following 30 minutes, 1 hour, or 3 hours of serum induction, were collected by filtration, washed, and lysed with glass beads in the Mini Beadbeater (Biospec Products, Bartlesville, OK) in extraction buffer containing 25mM HEPES-KOH, pH7.5/ 150mM KCl/ 2mM MgCl2/0.1% Nonidet P-40/1mM DTT/ 200U/ml RNasin/1mM each of PMSF and benzamidine/5 g/ml aprotinin/200 μg /ml amino-benzamidine/100 μg /ml amino capriotic acid/ and 1 μg /ml each of leupeptin, pepstatin, and bestatin. Lysates were incubated with IgG-sepharose beads in the above buffer containing 200 μg /ml heparin. The immunoprecipitate was released from the beads by cleavage with TEVprotease (Invitrogen), and RNA was isolated by phenol-chloroform extraction followed by ethanol precipitation. For a mock RNA

immunoprecipitation, the *SHE3*-TAP parental strain SE6 was subjected to the same methods. Total RNA from the *SHE3*-TAP strain was harvested from cultures prepared as above and was isolated by a hot phenol protocol [9]. cDNA samples were derived from 8 μg of each RNA sample. Labeled cDNA was generated, coupled to fluorescent dyes and hybridized to DNA microarrays essentially as described [10]. Arrays were scanned on a GenePix 4000 scanner (Axon Instruments, Foster City), and data were quantified using GENEPIX PRO version 3.0 or 5.0 and were further processed using NOMAD (http://ucsf-nomad.sourceforge.net/). Processed data were analyzed in Microsoft Excel; filters applied to the data are described in the Results. cDNA samples from generated from total RNA from SE25 and SE6 were directly compared on DNA microarrays using the above methods. One transcript, *ECE1*, which was enriched in the SE25 strain, was eliminated from consideration as being She3-associated based on this strain difference.

Fluorescent *in situ* hybridization (FISH)

A template for each FISH probe was generated by cloning a portion of the corresponding gene into the pCR2.1 plasmid (Invitrogen, Carlsbad, CA) using primers listed in Supporting Table S2. Gene fragments were excised from the multiple cloning region and purified by phenol-chloroform extraction, and digoxigenin-labeled antisense riboprobes were generated from 500-600 ng DNA using the DIG RNA labeling kit and T7 RNA polymerase (Roche, Indianapolis, IN) from promoter sequences incorporated into 3' primers. Following a two-hour *in vitro* transcription reaction and DNase-treatment to digest template DNA, the probes were hydrolyzed by incubation with 125mM sodium carbonate, pH10.2, for 10-20 minutes at 65°C then ethanol-precipitated

and resuspended in 50-80ul buffer comprising 50% formamide/50% TE/ 0.1% Tween-20. Yeast and hyphal cells were grown as described above and fixed in 5% formaldehyde for 1 hour. Cells were washed and spheroplasted for 20-50 minutes in buffer comprising 1.2M sorbitol, 0.1M KPO_4, pH7, 0.2U/ul RNasin, and, for yeast, 40 μg /ml zymolyase 100T and 30mM beta mercaptoethanol, or, for hyphae, 80 μg /ml zymolyase and 60mM beta mercaptoethanol, then fixed for two minutes in 4% paraformaldehyde directly on the slide. Probe hybridization and signal detection with the HNPP Fluorescent Detection Set (Roche) were performed essentially as described [11], except that Triton X-100 replaced CHAPS in the hybridization buffer. Cells were mounted in FluorMount-G (SouthernBiotech, Birmingham, AL) containing 0.5 μg /ml DAPI and imaged on the Axiovert-200 (Carl Zeiss, Thornwood, NY).

REFERENCES

1. Wilson RB, Davis D, Enloe BM, Mitchell AP (2000) A recyclable Candida albicans URA3 cassette for PCR product-directed gene disruptions. Yeast 16: 65-70.
2. Fonzi WA, Irwin MY (1993) Isogenic strain construction and gene mapping in Candida albicans. Genetics 134: 717-728.
3. Noble SM, Johnson AD (2005) Strains and strategies for large-scale gene deletion studies of the diploid human fungal pathogen Candida albicans. Eukaryot Cell 4: 298-309.
4. Liu H, Kohler J, Fink GR (1994) Suppression of hyphal formation in Candida albicans by mutation of a STE12 homolog. Science 266: 1723-1726.
5. Mitrovich QM, Tuch BB, Guthrie C, Johnson AD (2007) Computational and experimental approaches double the number of known introns in the pathogenic yeast Candida albicans. Genome Res 17: 492-502.
6. Inglis DO, Johnson AD (2002) Ash1 protein, an asymmetrically localized transcriptional regulator, controls filamentous growth and virulence of Candida albicans. Mol Cell Biol 22: 8669-8680.
7. Rigaut G, Shevchenko A, Rutz B, Wilm M, Mann M, et al. (1999) A generic protein purification method for protein complex characterization and proteome exploration. Nat Biotechnol 17: 1030-1032.
8. Longtine MS, McKenzie A, 3rd, Demarini DJ, Shah NG, Wach A, et al. (1998) Additional modules for versatile and economical PCR-based gene deletion and modification in Saccharomyces cerevisiae. Yeast 14: 953-961.

9. Miller MG, Johnson AD (2002) White-opaque switching in Candida albicans is controlled by mating-type locus homeodomain proteins and allows efficient mating. Cell 110: 293-302.
10. Bennett RJ, Uhl MA, Miller MG, Johnson AD (2003) Identification and characterization of a Candida albicans mating pheromone. Mol Cell Biol 23: 8189-8201.
11. Takizawa PA, Sil A, Swedlow JR, Herskowitz I, Vale RD (1997) Actin-dependent localization of an RNA encoding a cell-fate determinant in yeast. Nature 389: 90-93.
